# Emotional appraisal in gameful work design: a conceptual framework for divergent employee reactions

**DOI:** 10.3389/fpsyg.2026.1906302

**Published:** 2026-09-25

**Authors:** Kaiyuan Zhao, Xiongying Niu

**Affiliations:** Business School, University of International Business and Economics, Beijing, China

**Keywords:** cognitive appraisal theory, double-edged effect, emotional appraisal, gameful work design, work gamification

## Abstract

Gameful work design is increasingly used to make tasks, feedback, performance systems, and workplace interaction more engaging, yet its consequences remain double-edged. Existing research has largely explained these differences in terms of motivation, technology acceptance, or performance outcomes, offering limited insight into why similar features are interpreted differently across employees and contexts. Drawing on cognitive appraisal theory and a bounded integrative evidence base of 52 publications, this conceptual analysis develops an emotional appraisal framework of gameful work design. The framework analytically distinguishes five interrelated appraisal domains—system characteristics, task design, self-evaluation and identity, social interaction, and organizational meaning—and explains how external design cues acquire supportive or threatening meanings through appraisal. These meanings shape emotional reactions, which may subsequently influence individual, team, and organizational outcomes. Eight propositions translate the framework into domain-focused, cross-pathway, multilevel, and temporal relationships for future testing. By clarifying what employees appraise and how appraisal processes link similar gameful features to divergent consequences, the framework extends cognitive-emotional explanations of work gamification. It provides a focused agenda for future research.

## 1 Introduction

Digital technologies, platform-based systems, and algorithmic tools are increasingly changing how employees experience work. In this context, digitized work environments have not only transformed task execution, performance management, and employee interactions but also increased employees’ expectations for feedback, participation, and meaningful work experiences. In response to these changes, organizations have begun to explore new forms of work design to enhance employee engagement and improve work experiences, making gamification an increasingly important approach. More broadly, gamification is commonly defined as the use of game design elements in non-game contexts (Deterding et al., 2011). In organizational settings, these elements are often introduced to increase task appeal, strengthen feedback, encourage employee participation, and support desired work behaviors (Cardador et al., 2017; Koivisto and Hamari, 2019).

Thus far, the organizational consequences of gameful work design are not uniformly positive. Studies have linked work gamification to stronger participation, learning, creativity, and performance (Gerdenitsch et al., 2020; Zhao and Huang, 2026), but also to work intensification, strategic compliance, resistance, and strained workplace relations (Hammedi et al., 2021; Vasudevan and Chan, 2022). Even the same mechanism may produce opposing outcomes: leaderboards may clarify goals and stimulate effort, but may also encourage avoidance, excessive competition, or non-cooperation (Landers et al., 2017). Work gamification is therefore a potentially double-edged intervention. The resulting question is why gameful work design produces constructive outcomes in some settings but dysfunctional outcomes in others.

Existing research has provided important insights into the effects of work gamification and has largely explained these differences through motivational or technology-acceptance perspectives. Motivational accounts typically propose that gameful design alters effort and persistence by satisfying or frustrating employees’ needs for autonomy, competence, and relatedness (Sailer et al., 2017; Mitchell et al., 2020). Technology-acceptance accounts focus on whether employees regard a gamified system as useful, easy to use, and worth continuing to use, and use these judgments to explain behavioral intentions, and subsequent behavior (Davis, 1989; Koivisto and Hamari, 2019). These perspectives help explain when gamification may be accepted, motivating, or effective in encouraging desirable employee behaviors (Miri and Macke, 2022). However, they provide limited insight into how employees interpret the meanings conveyed by gameful designs and why similar features generate divergent emotional and behavioral reactions. Increased participation or improved performance therefore cannot be assumed to indicate genuine acceptance, because such outcomes may also reflect compliance with rewards, rankings, or managerial expectations. These perspectives also say less about how employees’ interpretations reshape workplace relationships and contribute to cooperation, conflict, trust, or resistance. Existing research thus lacks an integrated explanation of how gameful features acquire emotional meaning and translate into broader employee and organizational consequences.

The emotional dimension is especially important because emotions reflect how employees experience the changes introduced by gamification and link those experiences to subsequent behavioral responses (Hammedi et al., 2021; Santabarbara et al., 2026). Mullins and Sabherwal (2020) advanced this line of inquiry by integrating the mechanics–dynamics–emotions framework with the cognitive structure of emotions. Their framework explains how game mechanics prompt individuals to evaluate anticipated outcomes, thereby linking them to specific emotional experiences (Robson et al., 2015). However, two issues remain insufficiently explained. First, the framework begins primarily from a gamification-design perspective and pays less attention to how employees appraise gameful design and the objects to which those appraisals are directed. It therefore struggles to explain why similar gameful designs elicit divergent employee reactions. Second, the framework focuses mainly on the relationship between game mechanics and emotions, but does not systematically explain how emotions further shape employee behavior and work outcomes. This limitation also constrains managerial understanding of the conditions required for effective implementation and the potential risks involved.

Cognitive appraisal theory provides the theoretical basis for addressing this gap. It holds that emotions are shaped not by external events alone, but by how individuals evaluate those events in relation to their goals, coping capacities, identities, and social contexts (Folkman et al., 1986). In gameful work design, employees therefore do not respond mechanically to gameful features; they interpret whether those features signal challenge, growth, recognition, and connection or instead control, surveillance, exposure, and competitive pressure. The same feature may consequently acquire different appraisal meanings and evoke different emotional reactions across employees and contexts. The central issue is thus not only which emotions gamification generates, but what employees evaluate, how appraisal meanings are formed, and how those meanings translate gameful features into emotional reactions and subsequent outcomes (Wei et al., 2025).

Based on the above discussion, this article attempts to develop an emotional appraisal framework of gameful work design from a cognitive appraisal perspective. The framework follows a four-part chain—gameful design features, employees’ appraisal meanings, emotional reactions, and subsequent behaviors and work outcomes—to explain why similar designs may generate divergent employee reactions. To specify where appraisal meanings arise, the article identifies five appraisal domains: system characteristics, task design, self-evaluation and identity, social interaction, and organizational meaning. These domains represent the primary objects employees evaluate rather than mutually exclusive categories of game elements. Through these domains, gameful features may be interpreted as supportive, meaningful, and relationship-enhancing in some contexts, but as pressuring, exposing, controlling, or unfair in others. On this basis, the article develops theoretical propositions and a research agenda for the future. This framework extends cognitive–emotional explanations of gameful work design and provides a basis for understanding the conditions for its implementation and potential risks.

## 2 Conceptual foundations

### 2.1 Gameful work design in organizational psychology

Existing research offers two influential definitions of gamification that differ in emphasis. Deterding et al. (2011) define gamification as the use of game design elements in non-game contexts, emphasizing the introduction of game-derived elements into activities that are not games. Huotari and Hamari (2017), from a service-marketing perspective, define gamification as enhancing a service through affordances for gameful experiences to support users’ overall value creation. The former takes game design elements as its starting point, whereas the latter places greater emphasis on the experiential possibilities afforded by design.

When gamification is applied to work settings, existing definitions have tended to emphasize the introduction of game design principles and elements. Oprescu et al. (2014) define workplace gamification as the adaptation and application of game design principles and game interaction elements to workplace processes and employee behavior. Cardador et al. (2017) view work gamification as a deliberate enhancement of traditional performance management systems, emphasizing its capacity to increase employees’ access to performance information and improve task experience. Accordingly, work gamification has often been understood as the organizational introduction of game design principles and elements into work processes and management systems. Still, the presence of these design elements alone does not determine employees’ experiences. From an affordance perspective, attention shifts to the possibilities these elements offer employees (Huotari and Hamari, 2017). In work settings, these possibilities may include opportunities for feedback, comparison, achievement display, interaction, and participation, and may acquire different meanings through employees’ appraisal processes.

On this basis, we use work gamification as an umbrella term for research on gamification in organizational and work contexts, and gameful work design as the focal construct to highlight specific organizational design arrangements. More specifically, we define gameful work design as a form of work design that incorporates game design elements into work and creates opportunities for employees to experience their work in gameful ways. In this definition, game design elements refer to the concrete design features introduced into work, whereas affordances capture the experiential possibilities these features make available to employees. Gameful features are therefore treated as external design cues for employee appraisal rather than as subjective experiences in their own right. The same design arrangement may lead to different work experiences and reactions depending on how employees appraise it.

### 2.2 Distinguishing gameful work design from related concepts

Gameful work design overlaps with, but is distinct from, playful work design and job crafting. All three may make work more enjoyable or meaningful, but they differ in how change is initiated and whether work content is altered. Playful work design is a bottom-up process in which employees actively create more fun or competitive work experiences by changing how they approach, frame, or enact work activities (Scharp et al., 2023). Job crafting also involves employee-initiated changes, but its scope is broader: employees may alter task boundaries, relational boundaries, or cognitive understandings of work better to fit their interests, values, and motivations (Bakker et al., 2020; Liu and Yin, 2024). Playful work design mainly changes how employees experience existing work, whereas job crafting may change the content or boundaries of work itself.

By contrast, gameful work design centers on gameful features embedded in work systems, task structures, feedback mechanisms, or organizational practices. These features are typically designed or introduced by organizations, managers, or system designers rather than initiated by employees. This distinction matters because employee-initiated playfulness and organization-designed gamification may carry different emotional meanings. A playful self-created challenge may feel autonomous and meaningful, whereas an organization-introduced leaderboard or point system may be appraised as either supportive recognition or managerial control.

However, organization-initiated design does not imply employee passivity. Employees may participate in design, adapt particular features, engage selectively, or resist specific arrangements, thereby shaping whether gameful work design is appraised as supportive and controllable or as imposed and controlling.

Accordingly, the present article does not treat all forms of play, fun, or self-directed work redesign as equivalent. Its focus is narrower: how employees appraise gameful features incorporated into organizational work design.

### 2.3 Employee emotions and emotional appraisal

Although gamification may elicit immediate emotional reactions through visual, auditory, and other experiential attributes, the sustained and divergent emotional consequences of gameful work design in organizational settings usually depend on how employees perceive and interpret these designs. The same gameful feature may carry different goal-related meanings for different employees: it may be viewed as a resource that supports work engagement and personal development, or as a means of increasing job demands or strengthening performance control. Gameful features therefore do not directly determine employees’ emotions in a fixed way; instead, they acquire specific emotional meaning through employees’ cognitive appraisals. Accordingly, this article treats emotional appraisal as the central psychological mechanism linking gameful work design to employees’ emotional reactions (Folkman et al., 1986; Mullins and Sabherwal, 2020).

Cognitive appraisal theory further explains how employees assign specific meanings to gameful events. In gameful work design contexts, employees typically interpret these events through different appraisal cues, with two important considerations concerning goal relevance and coping capacity. On the one hand, employees consider whether a gameful task is relevant to the work goals they value. When gameful design is seen as supporting competence development or work progress, employees may view it as an opportunity for growth and experience enthusiasm, hope, or pride. When it is seen as misaligned with personal goals or as creating additional burdens, it may instead be appraised as a threat or loss and evoke anxiety or disappointment. On the other hand, employees also consider their own capabilities and perceived control over outcomes when judging whether they can cope with gameful demands. Thus, even when facing the same gameful feature, employees may develop emotional reactions that differ in both nature and expression because they interpret the event’s meaning differently (Folkman et al., 1986; Ma et al., 2021).

More broadly, analyses of the emotional consequences of gameful work design should not stop at a binary distinction between positive and negative emotions. Valence and arousal describe the basic structure of emotional experience: valence reflects pleasantness, whereas arousal reflects activation (Russell, 1980; Watson and Tellegen, 1985). Still, emotions with the same valence may arise from different appraisal processes and have different implications. Anxiety, for example, often reflects appraisals of potential threat, uncertainty, and low control, and may prompt employees to invest more effort, seek information, or take preventive action to reduce uncertainty. Disappointment, by contrast, more often follows appraisals that goals have not been achieved or that outcomes fall short of expectations, and may lead to reduced investment or withdrawal from study. This article therefore focuses on discrete emotions, using valence and arousal only as supplementary concepts to describe structural differences among emotions rather than as dimensions in the subsequent analysis. Although cognitive appraisal theory explains how gameful features acquire emotional meaning, it remains necessary to identify the specific cues and sources that employees rely on when evaluating gameful work design.

### 2.4 Conceptual approach and evidence base

This conceptual analysis develops a framework explaining how employees appraise gameful work design and why their emotional reactions diverge. To support this conceptual development, we draw on a bounded integrative evidence base. Through a structured process of literature identification, screening, and synthesis, we identify core concepts and theoretical relationships from prior research.

The evidence base was developed from a focused search of the Web of Science database. The search covered publications from 2011 to 2026, reflecting the period after gamification became widely discussed as a formal research concept. More specifically, the search was conducted in the Web of Science Core Collection in July 2026. The search strategy combined gamification-related terms with terms referring to work and organizational contexts, including platform-based and algorithmically managed settings, as well as terms capturing emotional, appraisal-based, psychological, attitudinal, and behavioral reactions. The search returned 258 records. To be eligible, a publication had to examine gamification as a central design feature, mechanism, or organizational practice in a work-related context and provide theoretical or empirical evidence concerning employee or applicant reactions. The complete search strategy, eligibility criteria, and screening procedure are reported in Supplementary Table 1a. Because the search strategy included terms that capture emotional and appraisal-related reactions, the resulting evidence base is intentionally focused on studies relevant to this analysis’s emphasis on emotion and appraisal.

Following title and abstract screening, the remaining records were assessed against the eligibility criteria. This process resulted in a formal analytical sample of 52 publications (Supplementary Table 2). Publications identified through citation tracking, Google Scholar, or searches for foundational theories were retained only as supporting references and were not included in the formal analytical sample. Studies were excluded if they focused primarily on non-work contexts, such as education or tourism; mentioned gamification only in passing; did not examine work- or employee-related reactions; or provided insufficient evidence to inform the conceptual framework (Supplementary Table 1b).

The retained literature was then integrated using a theory-guided integrative approach. Cognitive appraisal theory informed the initial analysis, while cross-study comparison was used to identify recurring patterns linking gameful features, employee perceptions, appraisal meanings, emotional reactions, and subsequent outcomes. Through this synthesis, five appraisal domains were identified. These domains are interrelated but analytically distinct, and they provide the basis for the emotional-appraisal framework developed in the following sections.

## 3 Five appraisal domains in gameful work design

Building on the appraisal mechanism discussed above, this section shifts the focus from how employees evaluate gameful features to what they primarily evaluate. The five appraisal domains proposed in this study were not predefined as a fixed taxonomy. Rather, they were developed through a theory-informed integration of recurring patterns in prior research concerning gameful features, employee perceptions, appraisal meanings, and emotional reactions (Suh et al., 2018). Cognitive appraisal theory provides the interpretive framework for understanding how employees assign emotional meaning to gameful features, whereas prior studies reveal the specific objects repeatedly involved in employees’ appraisal processes. Based on this integration, this study identifies five major appraisal domains through which employees form emotional meanings in gameful work design. Conceptually, concrete gameful features function as external design cues, whereas the five appraisal domains specify the primary objects toward which appraisal is directed. Employees then judge those cues within one or more domains using criteria such as goal congruence, controllability, coping potential, fairness, identity consistency, and value congruence; the resulting interpretation constitutes appraisal meaning and subsequently evokes discrete emotions.

On this basis, we identify five appraisal domains, understood as analytically distinct objects of appraisal: system characteristics, task design, self-evaluation and identity, social interaction, and organizational meaning. System characteristics concern the interface, usability, aesthetics, narrative, and technical experience of the gameful system itself. Task design concerns how gameful mechanisms reshape work goals, challenges, progress, feedback, and task demands. Self-evaluation and identity concern what gameful information communicates about employees’ competence, contribution, achievement, status, and identity. Social interaction concerns how gameful mechanisms alter cooperation, competition, comparison, belonging, and workplace relationships. Organizational meaning concerns what employees infer about organizational values, authenticity, and managerial motives from the organization’s use of gameful work design. These domains distinguish the primary object of appraisal rather than the objective form of a game element.

The five domains extend analytically from concrete, proximal design experiences to broader organizational meaning: employees may evaluate the system they directly encounter, the work tasks reorganized by gameful mechanisms, what gameful information communicates about them, how it shapes relations with others, and what it reveals about the organization. This extension does not imply a fixed temporal sequence or causal hierarchy. The five domains distinguish the primary objects toward which appraisal is directed rather than assigning gameful features to mutually exclusive categories. Accordingly, a gameful feature does not have a fixed domain membership; it may primarily provide cues for one domain or, under certain conditions, for multiple domains, depending on how it is presented and what employees focus on. The following subsections therefore analyze each of the five domains through their central appraisal objects and distinctive emotional logics.

### 3.1 System characteristics

In a gameful work system, the interface, interactive feedback, and virtual narrative may provide cues for appraising the system’s experience. A clear and intuitive interface and timely, stable feedback can reduce the effort required to understand and operate the system. When these characteristics are appraised as easy to use and supportive of work goals, employees are more likely to experience enjoyment (Gerdenitsch et al., 2020). Coherent narratives and aesthetic design may similarly be appreciated when they are perceived as interesting and congruent with the work context (Suh et al., 2017). These reactions therefore depend on employees’ judgments of system usability, usefulness, and experiential appeal rather than on design characteristics alone (Bitrián et al., 2024).

However, complex operating procedures and unstable or delayed feedback may be perceived as difficult to use or as obstructing task completion, making frustration more likely (John et al., 2025). Research on gig work and virtual work training similarly suggests that reduced perceived autonomy under algorithmic management (Liu and Yin, 2024), excessive prompts, poorly integrated functions, or intrusive monitoring may reduce employees’ sense of control, disrupt established workflows, or demand additional attention, leading employees to appraise the system as an additional burden and intensifying frustration or annoyance (Bitrián et al., 2023; Eger et al., 2025).

### 3.2 Task design

Goal setting, progress feedback, and task rewards embedded in gameful mechanisms provide cues for appraising task clarity, progress, and attainability (Liu et al., 2018; Landers et al., 2017). Progress bars, levels, and badges can provide clear and timely feedback on current progress and task completion. Narrative can also serve as a task-design cue by situating task goals and progress within a coherent, work-related context, thereby making them more meaningful (Seo et al., 2021). When successful completion appears attainable, and tasks align with work goals, employees are more likely to appraise them as work-relevant and meaningful rather than as an additional burden, eliciting hope, joy, or excitement (Cardador et al., 2017; Passalacqua et al., 2020; Torresan and Hinterhuber, 2023).

By contrast, short deadlines and continuously updated performance requirements may be appraised as additional time pressure, eliciting anxiety or fear (Hammedi et al., 2021; Wei et al., 2025). Repetitive operations or compulsory participation may reduce perceived choice, turning an initially enjoyable design into a mechanical requirement and eliciting boredom or annoyance (Eger et al., 2025). More broadly, consistent with the job demands-resources (JD-R) perspective, gameful task features may add demands through performance pressure (Santabarbara et al., 2026), while progress feedback can serve as a resource for monitoring task progress (Bakker and Demerouti, 2017; Gerdenitsch et al., 2020). When task demands exceed available abilities and resources, employees are more likely to appraise them as threats rather than challenges, which can elicit frustration or anxiety. Thus, the emotional meaning of gameful tasks depends on how employees understand the relationship between task demands and their own resources (Wei et al., 2025).

### 3.3 Self-evaluation and identity

When employees focus on what gameful information communicates about themselves, leaderboards, points, and virtual identities become direct cues for evaluating competence, contribution, status, and identity. Badges may also take on self-evaluative meaning when they are interpreted as signals of personal competence, achievement, or contribution rather than merely as markers of task progress. Visible gameful indicators can help employees recognize progress and contribution; when higher levels, badges, or rankings are interpreted as recognition of effort, they are more likely to elicit excitement (Landers et al., 2017; Bizzi, 2023). Greater choice over which achievements, skills, or identities to display, together with consistency between these representations and employees’ abilities and identity, may strengthen feelings of recognition and elicit pride (Barbosa and Faria Santos, 2026).

Conversely, public leaderboards, real-time, quantified performance indicators, and displays with limited privacy controls may be perceived as exposure rather than recognition. The effects of real-time quantification may vary with work complexity because visible indicators may represent performance and contribution less accurately in complex work (Bizzi, 2023). When indicators appear incomplete, repeatedly expose perceived shortcomings, or allow little control over what is displayed, they may threaten competence and self-image, eliciting shame or anxiety (Van Zoonen, 2026; Cameron et al., 2025).

### 3.4 Social interaction

Gameful work design often incorporates team tasks or social systems, such as liking functions, peer comments, and shared challenges, prompting employees to appraise their relationships with coworkers. When responsibilities are clearly allocated and members receive timely feedback on their contributions, employees are more likely to interpret the interaction as supportive and experience joy or gratitude (Kwak et al., 2019; Khan et al., 2024). When peer feedback communicates acceptance, or moderate competition is interpreted as shared participation rather than zero-sum confrontation, employees may also experience joy because their relationships with coworkers are affirmed (Morschheuser et al., 2017).

However, public comparison and competition for limited resources may be perceived as zero-sum, exclusionary, or unfair, thereby creating a relationship threat. When interaction rules turn coworkers from collaborators into direct threats, employees are more likely to experience anxiety or envy (Van Zoonen, 2026). Even cooperative tasks may be appraised as unfair when they involve forced interdependence, unequal task allocation, or imbalanced recognition, thereby eliciting anger. Research on organizational socialization reports weak or mixed relationships between gamification, social integration, and competition (Peciura and Zukauskaite, 2024).

### 3.5 Organizational meaning

When employees focus on what gameful work design signals about the organization, organizational narratives and other symbolic design cues become important for appraising organizational values, authenticity, and managerial motives (Georgiou, 2021; Georgiou et al., 2019; Buil et al., 2020). In this context, a narrative that shapes system experience or task meaning may also be interpreted as expressing organizational values or managerial intentions. When these signals align with actual management practices and convey growth, recognition, and employee support, employees are more likely to appraise the design as evidence of authentic organizational support and to experience organizational pride (Schmidt et al., 2023; Ferreira et al., 2026).

However, compulsory participation, the use of playful framing to package additional work demands, opaque data-use rules (Zhang et al., 2025), and inconsistencies between organizational messaging and actual management practices may lead employees to question the real purpose of gamification (Popan et al., 2023). A recent study of platform work suggests that narratives emphasizing autonomy and self-direction may coexist with invasive algorithmic control (Cameron et al., 2025). Under such conditions, when employees appraise gamified arrangements as serving monitoring and performance control rather than employee support, they may experience disappointment or anger.

The five appraisal domains and their corresponding appraisal objects, meanings, and emotional reactions are summarized in Table 1.

**Table 1:** Five appraisal domains in gameful work design.

Primary appraisal domain	Illustrative gameful design cues	Evaluation focus (primary appraisal object)	Supportive appraisal pathway (meaning → emotion)	Threat appraisal pathway (meaning → emotion)
System characteristics	Interfaces, avatars, narratives, virtual environments, interactive feedback, aesthetic and audiovisual design.	Whether the gameful system itself is easy to use, controllable, appealing, and supportive of work.	The system is usable, controllable, and supportive of work goals → curiosity and enjoyment.	The system is complex, intrusive, or burdensome → frustration and annoyance.
Task design	Points, badges, progress indicators, levels, narratives, challenges, timed tasks, goals, rewards	How gameful mechanisms shape work goals, challenge, progress, and task completion.	Tasks are clear, attainable, meaningful, and mastery-oriented → excitement and hope.	Tasks are time-pressured, unfair, unattainable, or repetitive → anxiety, anger, or boredom.
Self-evaluation and identity	Rankings, leaderboards, levels, experience points, skill trees, badges, achievement displays, virtual identity displays	What gameful information communicates about employees’ competence, contribution, achievement, status, and identity.	Performance indicators signal recognition, affirmation of competence, and identity consistency → pride.	Visibility creates public exposure, unfavorable comparison, and self-threat → shame and anxiety.
Social interaction	Social systems, peer feedback, cooperative tasks, competitive tasks, team challenges, shared goals, rankings, leaderboards	How gameful arrangements shape support, comparison, cooperation, and relationships with coworkers.	Interaction is inclusive, cooperative, and eliciting belonging → gratitude and joy.	Interaction is zero-sum, exclusionary, or unfair → envy and anger.
Organizational meaning	Narratives, culture-themed rewards, mission-linked quests, symbolic titles	What the organization’s use of gameful work design reveals about its values, authenticity, and managerial motives.	Gameful work design signals authentic organizational support and value congruence → organizational pride.	Gameful work design signals monitoring, control, manipulation, or value incongruence → disappointment or anger.

The primary appraisal domain organizes rows. Bolded cues indicate design features that may serve as appraisal cues across multiple domains; the examples are illustrative rather than exhaustive.

## 4 Integrative emotional-appraisal framework

The five appraisal domains clarify how employees assign emotional meaning to gameful work design. However, these domains should not be understood as separate categories operating in isolation. Accordingly, this section proposes an emotional appraisal framework for gameful work design that links gameful work design features, appraisal meanings, employee emotional reactions, and individual, team, and organizational outcomes. The framework treats external design cues, appraisal domains, appraisal criteria, appraisal meanings, and emotional reactions as conceptually distinct levels.

The framework explains the double-edged effect of gameful work design by shifting attention from game elements themselves to employees’ interpretations of what these elements mean in organizational contexts (Murawski, 2021). Drawing on cognitive appraisal theory and cognitive-emotional views of gamification, the framework treats gameful features as appraisal cues through which employees interpret tasks, feedback, visibility, social comparison, and organizational signals (Mullins and Sabherwal, 2020; Robson et al., 2015). Because their emotional meanings are not fixed, the same feature may initiate different pathways across employees or contexts.

Following cognitive appraisal theory, the framework assumes that employee emotions emerge when individuals evaluate work events in relation to their goals, values, identities, coping resources, and social position (Folkman et al., 1986). In gameful work design, concrete gameful features provide appraisal cues, whereas the five appraisal domains identify the domains or objects toward which evaluation is directed. Taken together, they show that employees evaluate both their immediate experience of performing work and the broader meanings that gameful work design carries for the self, workplace relationships, and the organization. Appraisals in the system-characteristics and task-design domains focus on usability, control, challenge, progress, and workload. In contrast, appraisals in the remaining three domains focus on recognition, identity, belonging, comparison, organizational values, and managerial intentions. Rather than constituting two new higher-order categories, these cues jointly establish the appraisal basis from which employees may construct supportive or threatening meanings; this process does not assume a fixed sequence from immediate experience to broader meaning. Employees judge these objects using criteria such as goal congruence, controllability, coping potential, fairness, identity consistency, and value congruence; the resulting interpretations constitute appraisal meanings.

In the supportive pathway, cues such as useful feedback, attainable challenge, visible recognition, relational connection, and value-congruent organizational signals are interpreted as facilitating work and advancing valued goals. This pathway does not involve a single undifferentiated positive effect. Clear progress and engaging task activity may evoke enjoyment or excitement; visible accomplishment and recognition of competence may evoke pride; and supportive social or organizational signals may evoke gratitude or a positive sense of belonging (Silic et al., 2020). Although these discrete emotions differ in their objects, they generally reinforce approach-oriented tendencies. Employees may remain engaged with the system (Robson et al., 2016), sustain effort, learn from feedback, help colleagues, and contribute constructively to task and team activities (Benitez et al., 2022; Yang et al., 2024). At the individual level, these responses may strengthen work engagement (Miri and Macke, 2022), learning (Liu and Zhou, 2025), wellbeing, self-efficacy, and task performance (Justin and Joy, 2023). Through repeated interaction, they may also enhance team cooperation (Rhee et al., 2020; Khan et al., 2024) and cohesion and, when the design is viewed as authentic and supportive, contribute to organizational identification and affective commitment (Getman et al., 2024).

In the threat pathway, cues such as burdensome demands, unattainable goals, public exposure, unfair comparison, surveillance, or managerial control are interpreted as placing valued goals, competence, social standing, or autonomy at risk. Difficult targets and excessive demands may elicit anxiety or frustration; unmet reward expectations may generate disappointment; and public rankings or unfavorable comparisons may evoke shame or envy (Burgoon et al., 2016). These emotions direct attention toward possible failure, exposure, or evaluation threat and consume resources needed for sustained work activity. Employees may consequently reduce voluntary participation, avoid difficult activities, share less information, cooperate less readily, or focus on preventing negative evaluation rather than improving the work itself (Novianto, 2025). At the individual level, these responses may increase strain and withdrawal and reduce task performance (Santabarbara et al., 2026); at the team level, they may weaken cooperation and foster conflict or distrust; and, when the design is viewed as manipulative, controlling, or value-incongruent, they may contribute to organizational alienation, cynicism, and resistance.

Beyond the possibility that a design cue may be relevant to more than one domain, the domains may also overlap within a particular appraisal when the same judgment simultaneously concerns appraisal objects from two or more domains. For ease of illustration, a public leaderboard provides a two-domain example of this form of overlap. In the self-evaluation and identity domain, employees may consider what their ranking communicates about their competence, achievement, and identity. In the social interaction domain, a public ranking places individual performance in relation to coworkers, drawing attention to employees’ relative position and its visibility to others (Van Zoonen, 2026). Furthermore, when employees use their relative position in public comparisons to judge their own competence or personal worth, the appraisal simultaneously concerns their competence and identity as well as their position in relationships with coworkers, thereby reflecting the overlap between the two domains. A lower ranking may signify inadequate competence and diminished group standing, combining threats to competence and social standing, evoking shame or anxiety, and prompting avoidance or reduced participation. Conversely, a higher ranking may signify affirmation of competence and social recognition, evoke pride, and support continued participation.

Cognitive appraisal theory is particularly well-suited as the organizing basis for this framework because it integrates processes that other perspectives typically address separately. Technology-acceptance perspectives can clarify system processes (Davis, 1989), while flow and job demands–resources perspectives illuminate complementary aspects of task processes, including challenge–skill fit and the balance between job demands and resources (Csikszentmihalyi, 2000); self-determination and social comparison perspectives can clarify need and comparison processes (Suh et al., 2018). Thus far, these perspectives generally focus on relatively specific appraisal objects or mechanisms. Cognitive appraisal theory provides a common language for explaining how heterogeneous cues from all five sources are interpreted in relation to goals, values, identities, resources, and social positions, how these interpretations evoke discrete emotions, and how those emotions extend into divergent work consequences. It therefore serves as the framework’s organizing mechanism without displacing these more local perspectives.

The framework therefore explains the double-edged effect of gameful work design as an appraisal-dependent process (Koivisto and Hamari, 2019; Zhao and Huang, 2026). Gameful features do not automatically produce positive or negative emotional reactions among employees. Their effects depend on how employees interpret the work-related, self-related, social, and organizational meanings attached to them. By integrating five appraisal domains with supportive and threat pathways, the emotional appraisal framework of gameful work design does not classify gameful features as inherently beneficial or harmful; rather, it explains how the same feature can generate divergent emotional reactions and work consequences across multiple levels through different appraisal meanings. Figure 1 synthesizes the preceding discussion.

**Figure 1:**
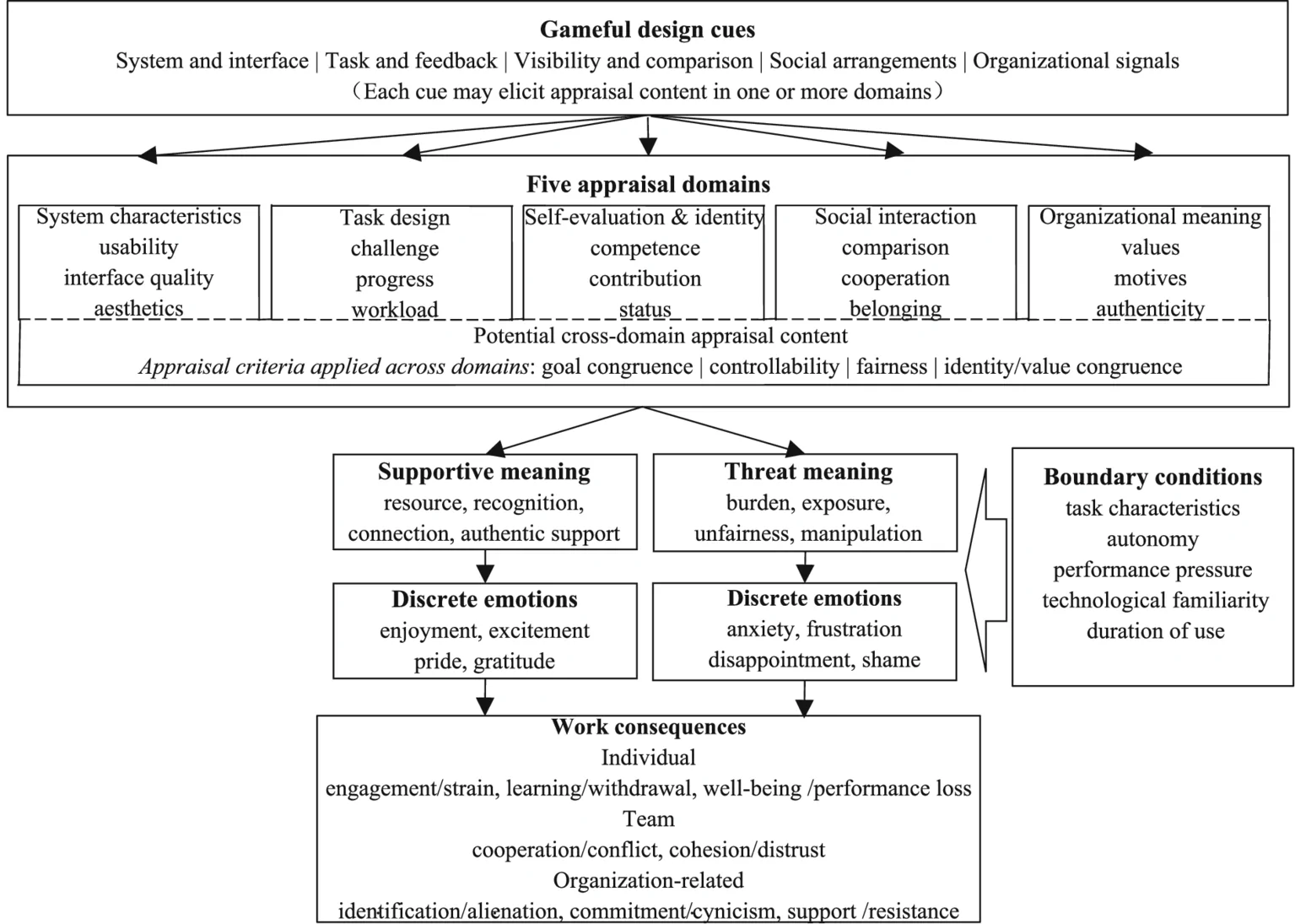
Emotional appraisal framework of gameful work design. The lower layer indicates that a particular appraisal may concern objects from two or more domains. Such overlap does not imply a common appraisal core or a fixed pattern of overlap among the five domains.

## 5 Propositions and future research directions

The integrative framework explains how gameful work design can generate divergent employee reactions through appraisal meanings. To move this framework toward future empirical examination, this section translates its core relationships into propositions. These propositions specify how different appraisal sources may shape employee emotional reactions and how these reactions may translate into individual- and team-level outcomes as well as broader organizational consequences. They are not intended as confirmed causal conclusions, but as theoretically grounded directions for future research.

The interface and feedback provided by a gameful system first shape whether employees can understand its rules and anticipate how the system will respond. A clear interface and timely feedback reduce information-search demands and cognitive load, making employees more likely to appraise the system as controllable and, in turn, to experience enjoyment. Greater predictability also helps employees maintain a steady pace of interaction and avoid unnecessary disruption (Davis, 1989; Suh et al., 2017). However, whether this sense of control is realized depends on employees’ autonomy in using the system. The positive effects should be stronger when employees can choose how they use the system, set their own pace, or adjust feedback settings (Van Roy and Zaman, 2019; Liu and Yin, 2024; Mao et al., 2024). When the system is experienced as both controllable and enjoyable, employees should be more willing to remain engaged with it. Accordingly, we propose Proposition 1.

Proposition 1: Clear interactive interfaces and immediate feedback increase employee enjoyment by strengthening employees’ appraisal of the gameful system as controllable, making continued engagement more likely; these appraisal and emotional responses should be stronger when employees have greater autonomy over system use.

Employees’ appraisals of levels, timed tasks, and progressively more difficult challenges depend on the relationship between task demands and their capabilities. When challenge and skill are well matched, employees experience the task as demanding yet attainable and believe that effort can lead to progress. They are therefore more likely to appraise the task as an opportunity for growth and to feel excited (Sailer et al., 2017; Mitchell et al., 2020). When challenge exceeds skill, employees may perceive that their available resources are insufficient to meet task demands, thereby strengthening threat appraisals and further evoking anxiety (Bakker and Demerouti, 2017; Wei et al., 2025). When skill exceeds challenge, insufficient stimulation makes it harder to sustain an absorbing task experience and is more likely to produce boredom (Csikszentmihalyi, 2000). Thus, different directions of challenge–skill misfit activate different appraisal pathways rather than producing the same negative response. These emotional reactions should also lead to distinct task-related responses: excitement can sustain engagement by reinforcing approach toward an attainable challenge, anxiety can increase strain by keeping attention focused on possible failure, and boredom can make withdrawal more likely when the task provides insufficient stimulation. Accordingly, we propose Proposition 2.

Proposition 2: When challenge and skill are well matched, gameful tasks are more likely to be appraised as opportunities for growth and to evoke excitement, which should help sustain task engagement; when challenge exceeds skill, tasks are more likely to be appraised as threatening and to evoke anxiety, which should increase strain; when skill exceeds challenge, tasks are more likely to be appraised as insufficiently stimulating and to evoke boredom, which should make work withdrawal more likely.

Points and leaderboards convert employee performance into publicly comparable information, whose meaning depends on employees’ relative performance position. For higher-performing employees, public results can affirm competence and achievement, making visibility more likely to be appraised as recognition and to evoke pride. Pride can motivate employees to maintain a favorable sense of competence and continue demonstrating their ability, thereby supporting continued participation. For lower-performing employees, public comparison draws attention to performance gaps and shortcomings, making visibility more likely to be appraised as threatening and to evoke shame. To protect the self from further unfavorable exposure, employees experiencing shame may become less willing to participate. The same visibility mechanism can therefore carry different meanings depending on where employees stand in the comparison (Morschheuser et al., 2017; Suh et al., 2018). Accordingly, we propose Proposition 3.

Proposition 3: In gameful settings where points and leaderboards make performance public, high relative performance is more likely to elicit competence-recognition appraisals and pride, with continued participation more likely to follow; low relative performance is more likely to elicit self-threat appraisals and shame, with participation more likely to decline.

Gameful cooperative tasks often involve shared goals, team points, or collective rewards, but their emotional and behavioral consequences depend on how employees interpret the relationship between others’ contributions and their own goals. When colleagues’ contributions help employees achieve both personal and team goals, employees are more likely to view those contributions as supportive and to feel gratitude (Riar et al., 2022). Gratitude can encourage employees to reciprocate this support, making continued cooperation more likely. When others’ success may reduce employees’ own rewards, opportunities, or status, it is more likely to be appraised as a threat to personal interests and to evoke envy (Morschheuser et al., 2017). Envy can reduce employees’ willingness to support those coworkers, making continued cooperation less likely. Accordingly, we propose Proposition 4.

Proposition 4: In gameful cooperative tasks, when colleagues’ contributions help advance both employees’ own goals and team goals, employees are more likely to form goal-support appraisals and feel gratitude, followed by greater cooperation; when colleagues’ success may reduce employees’ own rewards, opportunities, or status, employees are more likely to form interest-threat appraisals and feel envy, followed by lower cooperation.

A gameful narrative does more than add a story to a task; it can translate an organization’s vision into a value signal that employees can experience. When the narrative’s goals align with the organization’s vision, employees are more likely to view gamification as an authentic expression of the values the organization claims to uphold, thereby evoking organizational pride. Employees also compare the narrative with everyday management practices. When performance evaluations, rewards, and management practices align with the stated vision, appraisals of authenticity and organizational pride should be stronger. Such consistency between words and actions also reduces suspicion that gamification is merely symbolic packaging (Kim and Werbach, 2016; Butler and Spoelstra, 2024). Organizational pride may further strengthen employees’ sense of connection to the organization, thereby increasing the likelihood of organizational identification. Accordingly, we propose Proposition 5.

Proposition 5: Alignment between the goals of a gameful narrative and the organization’s vision is more likely to be appraised as an authentic expression of organizational values and to evoke organizational pride, with stronger organizational identification more likely to follow; these appraisal and emotional responses should be stronger when the organization’s stated vision is consistent with its day-to-day management practices.

Gameful work design often brings rewards, rankings, task challenges, and performance displays together within the same setting. Employees may interpret such a design as both an opportunity for growth and as a potential source of failure or loss. When supportive and threat appraisals are both strong, employees may simultaneously want to approach the gameful task and worry about the consequences of participation, thereby experiencing emotional ambivalence. Emotional ambivalence refers to the simultaneous experience of opposing emotions toward the same object, such as feeling both anticipation and concern or wanting both to approach and to avoid (Van Roy and Zaman, 2019). Because neither of these opposing action tendencies readily becomes dominant, employees must repeatedly weigh the benefits and risks of participation (Vasudevan and Chan, 2022). They are therefore more likely to delay making a decision and to hesitate about whether to take part. Accordingly, we propose Proposition 6.

Proposition 6: When the same gameful arrangement simultaneously elicits strong supportive and threat appraisals, employees are more likely to experience emotional ambivalence; the stronger this ambivalence, the more likely employees are to hesitate when deciding whether to participate in the gameful task.

Gameful arrangements built around shared goals and common rules do not necessarily produce shared appraisals. What matters is how team members understand the relationship between collective and individual goals. Shared goals create repeated interaction, and through information exchange, mutual observation, and emotional contagion, members may gradually adjust their judgments. Initially divergent individual appraisals may therefore become more aligned and develop into a shared team-level appraisal (Rhee et al., 2020; Khan et al., 2024). When sharing resources helps members achieve both personal and team goals, they are more likely to appraise the gameful task as an opportunity for mutual support and collective goal attainment, gradually developing a shared supportive appraisal. When resource sharing may weaken personal returns or increase competitive pressure, a shared threat appraisal is more likely to emerge. Shared supportive appraisals can foster a positive team emotional climate, reduce concerns about sharing experience and expressing ideas, and thereby promote knowledge sharing (Peciura and Zukauskaite, 2024). Shared threat appraisals, by contrast, can create a tense and defensive emotional climate in which members worry that sharing knowledge will erode their advantage, making them more inclined to withhold or conceal information (Liu and Hu, 2026). Accordingly, we propose Proposition 7.

Proposition 7: When team members develop shared supportive appraisals of a gameful arrangement, a positive team emotional climate is more likely to emerge and, in turn, promote team knowledge sharing; when they develop shared threat appraisals, a negative team emotional climate is more likely to emerge and, in turn, increase team knowledge hiding.

The emotional meaning of gameful work design is not fixed but may change with continued use. During initial use, the novelty of gameful features may lead employees to appraise the design as an opportunity to explore new approaches and gain new experiences, evoking curiosity and encouraging them to learn the system’s rules and try new ways of performing tasks. As use continues, employees become familiar with the rules and feedback mechanisms, and the novelty-related stimulation may fade. When gameful arrangements continue to rely on the same task routines, feedback formats, and interaction patterns over time, employees may cease to see them as opportunities for growth and instead appraise them as repetitive work demands, leading to boredom and a lower willingness to remain engaged (Cardador et al., 2017; Hammedi et al., 2021). Accordingly, we propose Proposition 8.

Proposition 8: The emotional meaning of gameful work design changes over time. In the short term, the novelty of gameful features makes employees more likely to appraise the design as an opportunity for exploration, thereby evoking curiosity and promoting exploratory learning; over the longer term, declining novelty may lead employees to appraise the design as a repetitive burden, evoking boredom and increasing work withdrawal.

Together, these propositions move the emotional appraisal framework into empirically testable relationships. The eight propositions are summarized in Table 2.

**Table 2:** Summary of propositions, appraisal mechanisms, and associated responses.

Proposition	Analytical focus	Gameful design feature or mechanism	Core appraisal	Emotional response	Subsequent work-related response
P1	System characteristics	Clear interactive interfaces and immediate feedback	Perceived controllability of the gameful system	Enjoyment	Continued system engagement
P2	Task design	Levels, timed tasks, and progressively more difficult challenges	Challenge–skill fit appraisal	Excitement; anxiety; boredom	Task engagement; strain; work withdrawal
P3	Self-evaluation and identity	Points and leaderboards that make relative performance visible	Competence recognition versus self-threat appraisal	Pride; shame	Continued/reduced participation
P4	Social interaction	Shared goals, team points, and reward structures	Goal support versus interest threat appraisal	Gratitude; envy	Greater/reduced cooperation
P5	Organizational meaning	A gameful narrative aligned with the organization’s vision	Authenticity appraisal of organizational values	Organizational pride	Organizational identification
P6	Multiple appraisal pathways	Combined rewards, rankings, task challenges, and performance displays	Supportive–threat appraisal conflict	Emotional ambivalence	Participation hesitation
P7	Social interaction—team-level extension	Shared goals and common rules, repeated interaction, and resource sharing	Shared supportive versus threat appraisal	Positive or negative team emotional climate	Team knowledge sharing; team knowledge hiding
P8	Temporal dynamics	Novel gameful features and continued exposure to recurring task routines, feedback formats, and interaction patterns	Novelty versus repetitive-burden appraisal	Curiosity; boredom	Exploratory learning; work withdrawal

P1–P5 represent domain-focused propositions corresponding to the five appraisal domains, whereas P6–P8 extend the framework through cross-pathway, multilevel, and temporal processes.

## 6 Discussion

This conceptual analysis shows that the effects of gameful work design depend on how employees interpret its meanings within different appraisal domains, including gameful system characteristics, their work, the self, workplace relationships, and the organizational context. The following discussion considers the broader theoretical and practical implications of this conceptual analysis.

### 6.1 Theoretical implications

First, this article develops an emotional appraisal framework that integrates a complete explanatory chain linking gameful design features, employees’ appraisal meanings, emotional reactions, and subsequent behaviors and work outcomes. Existing research has mainly examined the effects of gameful design from motivational and technology-acceptance perspectives, but has not systematically explained how employees’ psychological processing of gameful features produces divergent outcomes. The framework therefore helps clarify the complex relationship between gameful work design and employee reactions.

Second, by identifying five appraisal domains grounded in the work context, this article advances the cognitive-emotional explanation of gamification. Mullins and Sabherwal (2020) show how game mechanisms can evoke emotional reactions through appraisals of event consequences, agents’ actions, and object attributes. Building on this account, the present article specifies the objects toward which employees direct their appraisals of gameful design in concrete work settings. Importantly, these domains are analytically distinct but non-exclusive: a single design cue may inform appraisals in more than one domain, while some appraisals may simultaneously concern multiple appraisal objects, thereby reflecting domain overlap. It thus contextualizes the general process through which appraisal elicits emotion by specifying the organizational objects around which employees construct emotional meaning, helping to explain why similar gameful features acquire different meanings across employees and organizational contexts.

Third, this article deepens understanding of the double-edged effects of gameful work design from a discrete-emotion perspective. Existing gamification research often treats employee emotions in broad positive-and-negative terms, with limited attention to the specific roles of discrete emotional states in the gamification process. By distinguishing among the discrete emotions elicited by gameful design and clarifying their different functional implications, this article provides a more fine-grained emotional account of why the same gameful design may promote constructive employee responses in some contexts but produce dysfunctional responses in others.

### 6.2 Practical implications

The framework also offers guidance for designing emotionally sustainable gameful work. First, organizations should move from a focus on game elements to a gameful design approach centered on employee appraisal. The effectiveness of gamification does not depend solely on whether points, badges, or leaderboards are used, but on how employees interpret these designs in relation to their goals, autonomy, competence, and relationship with the organization (Kim and Werbach, 2016; Butler and Spoelstra, 2024). Before implementation, organizations should consider employee needs and work contexts, pilot-test gameful arrangements to identify unintended appraisal and emotional consequences, and use participatory design and clear communication to reduce negative interpretations.

Second, organizations should attend to employees’ emotional experiences rather than focusing only on performance outcomes. Points, rankings, and performance displays may strengthen feedback and feelings of achievement, but they may also create pressure, exposure, and competitive conflict. Organizations should therefore make data-use rules, evaluation criteria, and the purpose of feedback explicit, avoid reducing complex work contributions to simple metrics, and consider how different employees may experience the same gameful design. Where feasible, employees should also be given reasonable opportunities to customize or opt out of particularly intrusive, publicly visible, or nonessential gameful features. Organizations should also consider accessibility and inclusion by offering adjustable pacing, accessible interface formats, and alternatives to publicly visible participation for employees with disabilities, limited digital familiarity, or different working conditions.

Third, organizations should establish mechanisms for ongoing evaluation and adaptive adjustment, treating gamification as a continuing governance process. Employees’ appraisals of gameful design may change over time in response to work pressure and team interactions, and positive short-term experiences do not necessarily indicate long-term effectiveness (Eger et al., 2025). Organizations should therefore continue to monitor employees’ emotions, trust, perceived fairness, and participation, and revise game mechanisms, goal settings, and information displays in response to emerging feedback, so that gameful design remains aligned with the employee experience over time.

### 6.3 Limitations and future research

This article is a conceptual analysis and does not provide an empirical test of the proposed framework. Future studies are needed to examine whether the proposed appraisal sources and appraisal meanings explain employee emotional reactions across different organizational settings.

First, the five appraisal domains proposed in this article were developed through a theory-informed integration of this bounded evidence base, and their empirical boundaries and possible alternative classifications remain to be examined. Because a particular gameful design may involve several appraisal objects simultaneously, the domains may overlap or operate in combination. Future research could use interviews, critical-incident methods, and scale development to examine their empirical distinctiveness, patterns of combination, relative salience, and variation across contexts, thereby refining the framework.

Second, the framework explains employee reactions through a relatively static process chain and leaves the dynamics of this process less fully developed. Employees’ appraisals and emotional experiences may change across stages of use, work experiences, and organizational interactions. Future research could combine experience sampling, longitudinal designs, and experiments to examine a cyclical process of appraisal, emotion, and reappraisal, and to compare short-term experiences with longer-term adaptation.

Third, although this article focuses on how employees appraise gameful work design, these appraisals may also vary with employee characteristics, agency, and work context. Future research could examine how autonomy needs, gaming experience, technological familiarity, participation, adaptation, selective engagement, and resistance shape perceived control, fairness, authenticity, and emotional reactions across cultural and occupational settings and under conditions such as remote or hybrid work, algorithmic management, and employment insecurity.

## 7 Conclusion

Gameful work design may evoke divergent emotional reactions among employees because they assign different appraisal meanings to gameful features in specific work contexts. More specifically, gameful work design features provide appraisal cues, whereas the five appraisal domains indicate the primary objects through which employees interpret these cues. To explain this process, this article develops an emotional-appraisal framework linking gameful work design features, appraisal meanings, discrete emotional reactions, and subsequent work outcomes. The framework shows that similar gameful features may activate supportive or threat appraisal pathways, thereby producing different emotional and behavioral consequences.

The framework identifies five appraisal domains: system characteristics, task design, self-evaluation and identity, social interaction, and organizational meaning. These domains clarify the primary objects through which employees assign emotional meaning to gameful work design, how different appraisals elicit employee emotional reactions, and how these reactions subsequently influence individual- and team-level outcomes as well as broader organizational consequences. By shifting attention from game elements themselves to employees’ emotional appraisals, this article offers a conceptual basis for understanding the double-edged effect of gameful work design in organizational psychology.
