# Supplementary material for: Emotional appraisal in gameful work design: a conceptual framework for divergent employee reactions
Source: Front Psychol. 2026 Sep 25;17:1906302. doi: 10.3389/fpsyg.2026.1906302 (PMC13649507; doi:10.3389/fpsyg.2026.1906302)
Supplement: Supplementary file 1 [file Supplementary_file_1.docx]

**Supplementary Material**

**Supplementary Table 1a. Search strategy**

| **Parameter** | **Specification** |
| --- | --- |
| Database | Web of Science Core Collection |
| Search date | July 2026 |
| Publication years | 2011–2026 |
| Search string | TS=((gamif* OR gameful) NEAR/3 (work* OR employee* OR worker* OR workplace* OR organization* OR organisation* OR "human resource*" OR "performance management" OR gig OR platform*)) AND TS=(emotion* OR affect* OR appraisal* OR enjoyment OR anxiety OR stress OR strain OR fatigue OR frustration OR pride OR shame OR envy OR trust OR distrust OR well-being OR wellbeing OR engagement OR satisfaction OR resistance) AND PY=(2011–2026) |
| Language and source type | English-language journal records indexed in the Web of Science Core Collection. Conference-only records were not included in the formal analytical sample. |

**Supplementary Table 1b. Screening summary and eligibility criteria**

| **Stage** | **Records (n)** | **Decision / criterion** |
| --- | --- | --- |
| Records identified | 258 | — |
| Title and abstract screening | 176 excluded; 82 retained | Excluded records outside work contexts, records mentioning gamification only peripherally, and records without work- or employee-related responses. |
| Full-text eligibility assessment | 30 excluded; 52 retained | Excluded records with insufficient work-context relevance, peripheral treatment of gamification, or insufficient substantive evidence for the analytical framework. |
| Final analytical sample | 52 | Publications included in the formal analytical sample are listed in Supplementary Table 2. |
| Inclusion criteria | — | Publications were retained when gamification was examined as a central design feature, mechanism, or organizational practice in a work-related context and when theoretical or empirical evidence concerning employees or applicants was provided. |

**Note.** The 52 publications constitute the formal analytical sample. Foundational theoretical, definitional, and contextual sources used for conceptual grounding were not included in this count.

**Supplementary Table 2. Publications Included in the Formal Analytical Sample**

| No. | Study | Relevant appraisal domain(s) |
| --- | --- | --- |
| 1 | Barbosa, B., & Faria Santos, A. (2026). The role of gamification in fostering employee involvement in innovation. European Journal of Innovation Management, 29(11), 254–274. | Self-evaluation and identity; Social interaction; Organizational meaning |
| 2 | Benitez, J., Ruiz, L., & Popovič, A. (2022). Impact of mobile technology-enabled HR gamification on employee performance: An empirical investigation. Information & Management, 59(4), 103647. | System characteristics; Task design |
| 3 | Bitrián, P., Buil, I., Catalán, S., & Hatfield, S. (2023). The use of gamification strategies to enhance employees’ attitudes towards e-training systems. The International Journal of Management Education, 21(3), 100892. | System characteristics; Task design |
| 4 | Bitrián, P., Buil, I., Catalán, S., & Merli, D. (2024). Gamification in workforce training: Improving employees’ self-efficacy and information security and data protection behaviours. Journal of Business Research, 179, 114685. | System characteristics; Task design |
| 5 | Bizzi, L. (2023). Why to gamify performance management? Consequences of user engagement in gamification. Information & Management, 60(3), 103762. | Self-evaluation and identity; Organizational meaning |
| 6 | Buil, I., Catalán, S., & Martínez, E. (2020). Understanding applicants’ reactions to gamified recruitment. Journal of Business Research, 110, 41–50. | System characteristics; Organizational meaning |
| 7 | Butler, N., & Spoelstra, S. (2024). Redemption through play? Exploring the ethics of workplace gamification. Journal of Business Ethics 193, 259-270. | Organizational meaning |
| 8 | Cameron, L. D., Conzon, V. M., & Lam, L. (2025). Selling the self: Neo-normative control and the platform paradox. Research in Organizational Behavior, 45, Article 100230. | Self-evaluation and identity; Organizational meaning |
| 9 | Cardador, M. T., Northcraft, G. B., & Whicker, J. (2017). A theory of work gamification: Something old, something new, something borrowed, something cool? Human Resource Management Review 27, 353-365. | Task design; Self-evaluation and identity; Organizational meaning |
| 10 | Eger, V. M., Georganta, E., Zuercher, P.-D. J., Mueller, F., Bohné, T., & Diefenbach, S. (2025). The power of play: Gamification in virtual workplace training. European Journal of Work and Organizational Psychology, 34(2), 218–236. | System characteristics; Task design |
| 11 | Ferreira, H., Roseira, C., & Patrício, R. (2026). Platform-based gamification to foster cross-organisational alignment—A case study. Creativity and Innovation Management, 35(1), 231–250. | System characteristics; Social interaction; Organizational meaning |
| 12 | Georgiou, K. (2021). Can explanations improve applicant reactions towards gamified assessment methods? International Journal of Selection and Assessment, 29(2), 253–268. | System characteristics; Organizational meaning |
| 13 | Georgiou, K., Gouras, A., & Nikolaou, I. (2019). Gamification in employee selection: The development of a gamified assessment. International Journal of Selection and Assessment, 27(2), 91–103. | System characteristics; Organizational meaning |
| 14 | Gerdenitsch, C., Sellitsch, D., Besser, M., Burger, S., Stegmann, C., Tscheligi, M., & Kriglstein, S. (2020). Work gamification: Effects on enjoyment, productivity and the role of leadership. Electronic Commerce Research and Applications, 43, 100994. | Task design; Self-evaluation and identity; Social interaction |
| 15 | Getman, A. P., Yaroshenko, O. M., Demenko, O. I., Lutsenko, O. Y., & Prokopiev, R. Y. (2024). Gamification for staff motivation: Impact on work efficiency and corporate culture at the international level. Journal of Economics and Management 46, 274-299. | Task design; Organizational meaning |
| 16 | Hammedi, W., Leclercq, T., Poncin, I., & Alkire, L. (2021). Uncovering the dark side of gamification at work: Impacts on engagement and well-being. Journal of Business Research 122, 256-269. | Task design; Self-evaluation and identity; Organizational meaning |
| 17 | John, A., Kwak, D.-H., Benitez, J., Zhao, Y., & Park, S. (2025). Determinants of gamification effectiveness: Perspectives of technology affordances and coping responses in the context of team-based gamified training. European Journal of Information Systems 34, 689-714. | System characteristics; Task design; Social interaction |
| 18 | Justin, E. M. A., & Joy, M. M. (2023). Exploring intrinsic motivating factors in gamified context: A mixed-method study. International Journal of Human-Computer Interaction, 39(19), 3728-3744. | Task design; Self-evaluation and identity; Organizational meaning |
| 19 | Khan, J., Zhang, Q., Zada, M., Saeed, I., & Khattak, S. A. (2024). Gamification in hospitality: Enhancing workplace thriving and employee well-being. Acta Psychologica 246, 104243. | Social interaction; Organizational meaning |
| 20 | Kim, T. W., & Werbach, K. (2016). More than just a game: Ethical issues in gamification. Ethics and Information Technology 18, 157-173. | Organizational meaning |
| 21 | Koivisto, J., & Hamari, J. (2019). The rise of motivational information systems: A review of gamification research. International Journal of Information Management 45, 191-210. | System characteristics; Task design |
| 22 | Kwak, D.-H., Ma, X., Polites, G., Srite, M., Hightower, R., & Haseman, W. D. (2019). Cross-level moderation of team cohesion in individuals' utilitarian and hedonic information processing: Evidence in the context of team-based gamified training. Journal of the Association for Information Systems, 20(2), 161-185. | System characteristics; Self-evaluation and identity; Social interaction |
| 23 | Landers, R. N., Bauer, K. N., & Callan, R. C. (2017). Gamification of task performance with leaderboards: A goal setting experiment. Computers in Human Behavior, 71, 508–515. | Task design; Self-evaluation and identity; Social interaction |
| 24 | Liu, M., Huang, Y., & Zhang, D. (2018). Gamification's impact on manufacturing: Enhancing job motivation, satisfaction and operational performance with smartphone-based gamified job design. Human Factors and Ergonomics in Manufacturing & Service Industries, 28(1), 38-51. | System characteristics; Task design; Self-evaluation and identity |
| 25 | Liu, R., & Yin, H. (2024). How algorithmic management influences gig workers’ job crafting. Behavioral Sciences, 14(10), 952. | Task design; Organizational meaning |
| 26 | Liu, S., & Zhou, H. (2025). Does gamified training improve training performance? A dual-pathway moderated mediation model. Journal of Business Research, 188, Article 115086. | System characteristics; Task design |
| 27 | Liu, W., & Hu, L. (2026). Promotion or prevention? Impact of gamified work on knowledge hiding among gig workers. Internet Research, 36(2), 472–493. | Task design; Self-evaluation and identity; Organizational meaning |
| 28 | Mao, M., Zhang, L., Li, J., & Rivkin, W. (2024). My job is a game, and I am the owner: How gamification facilitates self-leadership for gig workers. Journal of Business Research, 183, 114877. | Self-evaluation and identity; Organizational meaning |
| 29 | Miri, D. H., & Macke, J. (2022). Gamification, motivation, and engagement at work: A qualitative multiple case study. European Business Review, 34(2), 263–276. | Task design; Organizational meaning |
| 30 | Mitchell, R., Schuster, L., & Jin, H. S. (2020). Gamification and the impact of extrinsic motivation on needs satisfaction: Making work fun? Journal of Business Research 106, 323-330. | Task design; Self-evaluation and identity |
| 31 | Mullins, J. K., & Sabherwal, R. (2020). Gamification: A cognitive-emotional view. Journal of Business Research 106, 304-314. | System characteristics; Task design; Social interaction |
| 32 | Murawski, L. (2021). Gamification in human resource management: Status quo and quo vadis. German Journal of Human Resource Management 35, 337-355. | Task design; Social interaction; Organizational meaning |
| 33 | Novianto, A. (2025). Gamification from below as by form of resistance: Algorithm control, precarity, and resistance dynamic of Indonesian gig workers. New Technology, Work and Employment, 40(3), 380–389. | Social interaction; Organizational meaning |
| 34 | Passalacqua, M., Léger, P.-M., Nacke, L. E., Fredette, M., Labonté-Lemoyne, E., Lin, X., Caprioli, T., & Sénécal, S. (2020). Playing in the backstore: Interface gamification increases warehousing workforce engagement. Industrial Management & Data Systems, 120(7), 1309-1330. | System characteristics; Task design; Self-evaluation and identity |
| 35 | Peciura, Z., & Zukauskaite, I. (2024). A preliminary investigation of the relationship between gamification and organizational socialization outcomes: Does gamification live up to its expectations? SAGE Open, 14(1), 21582440231216524. | Social interaction; Organizational meaning |
| 36 | Popan, C., Perez, D., & Woodcock, J. (2023). Cards against gamification: Using a role-playing game to tell alternative futures in the gig economy. The Sociological Review 71, 1058-1074. | Social interaction; Organizational meaning |
| 37 | Riar, M., Morschheuser, B., Zarnekow, R., & Hamari, J. (2022). Gamification of cooperation: A framework, literature review and future research agenda. International Journal of Information Management 67, 102549. | Social interaction |
| 38 | Robson, K., Plangger, K., Kietzmann, J. H., McCarthy, I., & Pitt, L. (2015). Is it all a game? Understanding the principles of gamification. Business Horizons 58, 411-420. | System characteristics; Task design |
| 39 | Robson, K., Plangger, K., Kietzmann, J. H., McCarthy, I., & Pitt, L. (2016). Game on: Engaging customers and employees through gamification. Business Horizons 59, 29-36. | System characteristics; Task design; Social interaction |
| 40 | Santabarbara, C., Royle, T., & Wei, W. (2026). “Beating the app” or Pyrrhic victories? Gamification, workaholism and FoMO in UK food-delivery platforms. New Technology, Work and Employment, 41(1), 116–127. | Task design; Self-evaluation and identity; Organizational meaning |
| 41 | Schmidt, C. V. H., Manske, J., & Flatten, T. C. (2023). Experience matters: The mediating role of gameful experience in the relationship between gamified competition and perceived innovation culture. Creativity and Innovation Management, 32(4), 551–567. | Social interaction; Organizational meaning |
| 42 | Seo, K., Fels, S., Kang, M., Jung, C., & Ryu, H. (2021). Goldilocks conditions for workplace gamification: How narrative persuasion helps manufacturing workers create self-directed behaviors. Human-Computer Interaction 36, 473-510. | Task design; Organizational meaning |
| 43 | Silic, M., Marzi, G., Caputo, A., & Bal, P. M. (2020). The effects of a gamified human resource management system on job satisfaction and engagement. Human Resource Management Journal 30, 260-277. | System characteristics; Organizational meaning |
| 44 | Suh, A., Cheung, C. M. K., Ahuja, M., & Wagner, C. (2017). Gamification in the workplace: The central role of the aesthetic experience. Journal of Management Information Systems 34, 268-305. | System characteristics |
| 45 | Suh, A., Wagner, C., & Liu, L. (2018). Enhancing user engagement through gamification. Journal of Computer Information Systems 58, 204-213. | System characteristics; Task design |
| 46 | Torresan, S., & Hinterhuber, A. (2023). Continuous learning at work: The power of gamification. Management Decision 61, 386-412. | Task design |
| 47 | Van Zoonen, W. (2026). From motivation to resentment: Gamification and the envy dilemma. Group & Organization Management. | Self-evaluation and identity; Social interaction |
| 48 | Vasudevan, K., & Chan, N. K. (2022). Gamification and work games: Examining consent and resistance among Uber drivers. New Media & Society, 24(4), 866-886. | System characteristics; Social interaction; Organizational meaning |
| 49 | Wei, W., Wang, X., Han, S., & Xiong, A. (2025). Does job gamification perception promote the job involvement of gig workers? The role of cognitive assessment and overwork: Evidence from Chinese delivery platforms. Personnel Review 54, 41-61. | Task design; Organizational meaning |
| 50 | Yang, Q., Zhou, A., Tang, C., & Deng, T. (2024). The dual effect of gamification on customer-oriented organizational citizenship behavior in gig economy: A study of the mechanisms and moderators. Current Psychology, 43(35), 28218–28230. | Task design; Social interaction; Organizational meaning |
| 51 | Zhang, Y., Li, L., Ma, X., Sun, J., Dong, Y., & Yang, Z. (2025). Invisible algorithms, visible outcomes: Roles of emotional labor and work gamification in gigs. Computers in Human Behavior, 169, 108681. | Self-evaluation and identity; Organizational meaning |
| 52 | Zhao, K., & Huang, L. (2026). The double-edged effect of work gamification on employee innovation behavior—The different role of insight experience and error management climate. International Journal of Human–Computer Interaction, 42(9), 6688–6700. | Task design; Self-evaluation and identity; Organizational meaning |

Note. Domain coding indicates the appraisal domain(s) that each study most directly informs. The five-domain framework is derived from the synthesis of the evidence base as a whole, and studies may inform more than one domain where appropriate.
